# Association between blood lead and blood pressure: a population-based study in Brazilian adults

**DOI:** 10.1186/s12940-017-0233-5

**Published:** 2017-03-14

**Authors:** Ana Carolina Bertin de Almeida Lopes, Ellen Kovner Silbergeld, Ana Navas-Acien, Rachel Zamoiski, Airton da Cunha Martins Jr., Alissana Ester Iakmiu Camargo, Mariana Ragassi Urbano, Arthur Eumann Mesas, Monica Maria Bastos Paoliello

**Affiliations:** 1 Graduate Program in Public Health, Center of Health Sciences, State University of Londrina - UEL, Londrina, Paraná Brazil; 2 Department of Environmental Health Sciences, Johns Hopkins Bloomberg School of Public Health, Baltimore, MD USA; 3 Radiation Epidemiology Branch of the Division of Cancer Epidemiology and Genetics National Cancer Institute, National Institutes of Health, Washington D.C., USA; 4 Graduate Program in Toxicology, Faculty of Pharmaceutical Sciences of University of São Paulo – USP, Ribeirão Preto, São Paulo Brazil; 5 Graduate Program in Health Sciences, Center of Health Sciences, State University of Londrina - UEL, Londrina, Paraná Brazil; 6 Department of Statistics, State University of Londrina - UEL, Londrina, Paraná Brazil; 7 Department of Public Health, State University of Londrina – UEL, Londrina, Paraná Brazil; 8 Department of Molecular Pharmacology, Albert Einstein College of Medicine, Bronx, NY 10461 USA

**Keywords:** Blood lead, Blood pressure, Hypertension, Population-based

## Abstract

**Background:**

Environmental lead exposure among adults may increase blood pressure and elevate the risk of hypertension. The availability of data on blood lead levels (BLL) in adult Brazilian population is scarce and population-based studies are important for screening the population exposure and also to evaluate associations with adverse health effects. The goal of this study was to examine the association of BLL with blood pressure and hypertension in a population-based study in a city in Southern Brazil.

**Methods:**

A total of 948 adults, aged 40 years or older, were randomly selected. Information on socioeconomic, dietary, lifestyle and occupational background was obtained by orally administered household interviews. Systolic blood pressure (SBP) and diastolic blood pressure (DBP) were measured according to the guidelines *VI Brazilian Guidelines on Hypertension*. BLL were measured by inductively coupled plasma mass spectrometry technique. Multiple linear and logistic regression models were performed to evaluate associations of BLL with SBP and DBP, and with the chance of hypertension and of elevated SBP and DBP.

**Results:**

The geometric mean of BLL was 1.97 μg/dL (95%CI:1.90-2.04 μg/dL). After multivariable adjustment, participants in the quartile 4 of blood lead presented 0.06 mm/Hg (95%CI, 0.04-0.09) average difference in DBP comparing with those in quartile 1. Participants in the 90th percentile of blood lead distribution had 0.07 mmHg (95% CI, 0.03 to 0.11) higher DBP compared with those participants in the 10th percentile of blood lead. The adjusted OR for hypertension was 2.54 (95% CI, 1.17-5.53), comparing the highest to the lowest blood lead quartiles. Compared with participants in the 10th percentile of blood lead, participants in the 90th percentile presented higher OR for hypertension (OR: 2.77; 95% CI, 1.41 to 5.46).

**Conclusion:**

At low concentrations, BLL were positively associated with DBP and with the odds for hypertension in adults aged 40 or older. It is important to enforce lead exposure monitoring and the enactment of regulatory laws to prevent lead contamination in urban settings.

## Background

Chronic lead exposure and accumulation in the body can lead to progressive health effects. Among these effects, increased blood pressure, which is associated with the onset of cardiovascular diseases, has been linked to lead exposure. The hypertensive effects of lead have been widely reported in workers exposed to high levels of the metal and by experimental studies in which animals were exposed to long-term high doses of lead. Under occupational conditions, the development of hypertension has been implicated as a possible consequence of the nephropathy caused by lead exposure. Studies have concluded that lead exposure is a risk factor for raised blood pressure and hypertension even in the general population [1–4].

Publications of NHANES (National Health and Nutrition Examination Survey) data have reported a decline of environmental lead exposure in the United States and consequent lower blood lead concentrations in the American population. While early studies had noticed positive and significant associations between blood lead and blood pressure, more recent studies have questioned the consistency of this association in view of the lower blood lead concentrations observed in the last published data [5–10]. A meta-analysis of the epidemiological studies available from 1980 to 2001 reported that a 2-fold increase in blood lead concentration was associated with a 1.0 mm Hg increase in systolic blood pressure and a 0.6 mm Hg increase in diastolic blood pressure [11]. Recent data have suggested that the effect sizes in the associations of blood pressure and blood lead (mean of 1.51 μg/dL) are small and inconsistent [6], or that a modest association was found [12].

Hypertension is a major risk factor for cardiovascular disease, which remains the leading cause of death in Brazil, representing about 13% of disability adjusted life years (DALYs) [13, 14]. Globally, raised blood pressure is the leading noncommunicable diseases risk factor, to which 13% of deaths are attributed. The prevalence of raised blood pressure in adults aged 25 years or older was about 40% in 2008, and is generally more prevalent in men than in women (39% for men and 32% for women) [15]. In Brazil, the prevalence is similar to other countries, occurring in 35.8% of men and 30% of women with general estimate of over 30% in both sexes, and prevalence increases with age (≥60 years old) [16–18].

In view of the low blood lead concentrations found in our study (1.97 μg/dL) and the divergent results published on literature, we aimed to examine the relationship of blood lead with blood pressure and hypertension in a population-based sample of Brazilian adults living in southern Brazil. Besides, few studies have evaluated the scientific evidence on blood lead and adverse health effects on the Brazilian general population. Our study focused on adults aged 40 years or older who, as compared to younger persons have increased risk factors for cardiovascular diseases, and also were probably exposed to higher concentrations of lead before 1985, when the withdrawal of tetraethyl lead in automotive gasoline occurred in Brazil [19].

## Methods

The study population included adults aged 40 years or older residents in an urban area in southern Brazil. In 2011, participants were randomly selected and took part in a household interview, anthropometric measurements and laboratory tests. Complete information of the study design and sampling has already been published [20, 21].

The accomplished study had a census based design, using data from the Population Count 2007, when the city of Cambé had a total of 92,888 people, of whom 30,710 (33.1%) were aged 40 or older (46% men and 54% women) [22]. All census tracts in the urban region were included in the study and the number of subjects to be interviewed in each tract was calculated proportionally to the amount of men and women aged 40 or over, and a quota of individuals, according to gender and age range, with five-year intervals was defined.

A total of 1180 (88.3%) of the selected persons completed the interview and 959 (81.3%) performed blood collection [20]. For the present analysis, we used data from 948 subjects who participated in the interviews, who had performed blood tests and had blood pressure measurements. Eleven participants missing information on blood pressure were excluded from this analysis.

The measurements were taken according to the VI Brazilian Guidelines on Hypertension, organized by the Brazilian Hypertension Society [23], by using the digital equipment Omron HEM 742. Measurements were obtained at both arms, and in case of difference, we used data from the arm with the highest value as a reference for subsequent measures. At least three measurements were performed with one minute interval between them, with the participant in a sitting position. The mean of the last two blood pressure measurements was considered the most accurate blood pressure. Participants were considered as hypertensive if any of the following criteria were present: a systolic blood pressure of 140 mm Hg or higher, a diastolic blood pressure of 90 mm Hg or higher, or self-reported use of antihypertensive medication. Systolic and diastolic blood pressures were also examined as separate dichotomous variables considering the same cutoff values.

Whole blood samples of approximately 5 ml were obtained by venipuncture after skin disinfection with alcohol 70%, collected in heparinized metal-free containers and kept under refrigeration until sent to the laboratory. Once in the laboratory, the samples were stored at −50 °C until being processed. Blood lead concentration was measured in duplicate by inductively coupled plasma mass spectrometry (ICP-MS) in the Adolfo Lutz Institute, São Paulo. To evaluate the accuracy of the results, the standard reference material Toxic Metals in Bovine Blood, from National Institute of Standards and Technology (NIST 966 levels 1 and 2) was used. The obtained results were: 1,315 ± 0.05 μg/100 ml (level 1) and 24.95 ± 0.07 μg/100 ml (level 2), showing good agreement with the certified values, 1,459 ± 0.013 μg/100 ml and 25.27 ± 0.22 μg/100 ml, respectively. The within-and-between batch precision were 4% and 6% for 1,459 μg/100 ml and 1% and 3% for 25.27 μg/100 ml, respectively. The detection and the quantification limits were obtained by 10 consecutive measurements of a blood sample with low lead level. These limits were calculated as three times and 10 times the standard deviation of those measurements. The results were 0.0029 and 0.0096 μg/100 ml for detection limit and quantification limit, respectively. Considering the dilution factor used for the sample preparation, the final limit of quantification was 0.20 μg/100 ml. Only one sample was below the limit of quantification and in this case the value of 0.20 μg/100 ml was assigned. All the analyses were performed in an ISO Class 7 cleanroom facility.

Information on sex, age (years), race, education, income class, occupation, smoking and alcohol consumption was obtained from each subject by the orally administered household interviews [21]. Race was categorized as white and non-white according to self-reported answers. Education was based on the self-reported number of years of education completed, (0–3, 4–7, 8–11 and 12 or more years of study). Income class was defined according to the Economic Classification Criterion Brazil (*Critério de Classificação Econômica Brasil - CCEB*) from the Brazilian Association of Research Companies [24]. This tool estimates the purchasing power and economic status of respondents, divided into classes A through E. For this study, this variable was categorized as A/B classes (corresponding to the higher income levels), C (medium income level) and D/E classes (related to the lower income levels). Occupation was queried in terms of potential lead exposure, based on self-reported current or former employment in any of the following of the lead using industries as defined by CNAE (National Classification of Economic Activities). These included: manufacture and recycling of batteries, secondary smelters, alloy production, electroplating, welding, PVC and other plastic manufacture and rubber recycling industry. Cigarette smoking was categorized as never, former and current smoker and alcohol consumption was categorized as either drinking or not drinking alcoholic beverages. Body mass index (BMI) information was obtained from the physical examination and calculated as weight in kilograms divided by the square of height in meters. Weight was measured with an electronic scale, with a precision of 0.1 kg and maximum capacity of 150 kg. The individuals were weighted barefoot, standing upright at the center of the scale platform and wearing light clothing. Height was measured with a portable stadiometer with a precision of 0.1 m and maximum length of two meters. The participants stood, barefoot, with their back turned to the vertical surface of the equipment and head positioned in the Frankfurt plane, with arms relaxed at the side of the body, with palms facing the thighs united heels, touching the vertical part of the stadiometer and medial edges spaced apart. The movable part of the stadiometer was elevated until touching the vertex, compressing the hair [21]. Current antihypertensive medication use was self-reported and participants were considered as taking these medications according with the VI Brazilian Guidelines of Hypertension [23]. The antihypertensive medications were shown to the interviewer at the moment of the interview. However, some of these medications as *carvedilol* (alpha-beta adrenergic blocker) and *spironolactone* (potassium-sparing diuretic), both prescribed for the treatment of congestive heart failure, and the medication *verapamil retard* (calcium channel blocker), used to treat cardiac arrhythmia, were not considered as current use of antihypertensive medication [25, 26]. In the case of diuretics use, a non-specific medication for the treatment of hypertension, the participant was considered to have hypertension depending on the self-reported question. For this, the following question was asked to the respondent: “Which of the following diseases have you ever been told by a doctor or other health professional (doctor, nurse, pharmacist, etc.) do you have or had?” Total cholesterol, high-density lipoprotein (HDL), triglycerides and glucose were performed using automated methodology to Dimension® RXL biochemical system model. The low-density lipoprotein (LDL) was calculated using the Friedewald equation [27].

For statistical analysis we used the software Stata [28] to perform descriptive and inferential statistical tests. Means, percentages and standard errors were estimated to describe the sample characteristics. Blood lead levels were left skewed and log transformed for analysis. Outcome variables, systolic and diastolic blood pressures were, respectively, inverse and log transformed to follow normal distribution. Multiple linear regression models were performed to examine associations of blood lead with systolic and diastolic blood pressures comparing those participants in quartiles 2 to 4 of blood lead levels with those in quartile 1. Multiple logistic regression analysis was used to evaluate the risk of hypertension also by categorizing blood lead in quartiles. We also performed Pearson correlation analysis to verify the correlations between systolic and diastolic blood pressure with blood lead levels.

Regression models were constructed based on a priori knowledge and biologic association with blood pressure (age, sex, antihypertensive medication use and blood lead log transformed). Other covariates were added to the model in two separated blocks: Model 1 – sex, age, race, income, education, antihypertensive medication and blood lead level; Model 2 – model 1 + total cholesterol, triglycerides, glycemia, smoking, alcohol consumption and body mass index. A third model was further adjusted for occupation status. These variables were included because they were significantly associated with blood pressure outcomes in at least one of the models performed before the inclusion of blood lead, considering a *p* < .20. Additionally, we performed regression analyses with only the subgroup that was not taking antihypertensive medication to elucidate if we would find significant changes in results. After running each model, the distribution of the residuals was tested for normality. Statistical tests with *p* value < .05 were considered statistically significant.

## Results

The overall geometric mean of blood lead level was 1.97 μg/dL (95% CI, 1.89–2.04), and the means for the blood lead quartiles ranged from 0.96 μg/dL to 4.21 μg/dL in the lowest and higher quartile, respectively. Blood lead levels were higher in men, in older participants (50 to 59 years old), in non-white, in smokers and drinkers, and in subjects with normal BMI. Participants currently or former employed in lead industries had higher blood lead levels (2.65 μg/dL; 95% CI, 2.31–3.05) than those not employed (Table 1).

**Table 1:** Blood lead levels (μg/dL) by participant characteristics

Characteristic	*n* (%)	Percentile 50th (median)	Interquartile range	Min – max values	Geometric means (95% CI)	*p*-Value^*a*^
Overall	948 (100)	1.94	1.44	0.46 – 45.62	1.97 (1.90, 2.04)	
Sex						
Male	421 (44.4)	2.46	1.72	0.65 – 45.62	2.59 (2.46, 2.73)	<0.001
Female	527 (55.6)	1.59	0.99	0.46 – 27.91	1.58 (1.51, 1.65)	
Age (years)						
40 – 49	386 (40.7)	1.83	1.46	0.48 – 27.91	1.84 (1.73, 1.96)	0.001
50 – 59	294 (31.0)	2.09	1.42	0.54 – 21.83	2.13 (2.00, 2.28)	
≥ 60	268 (28.3)	1.94	1.36	0.46 – 45.62	1.98 (1.84, 2.12)	
Race						
White	562 (59.3)	1.87	1.42	0.46 – 45.62	1.88 (1.79, 1.97)	0.084
Non white	386 (40.7)	2.03	1.48	0.49 – 24.49	2.09 (1.97, 2.23)	
Education (years)						
0 – 3	229 (24.2)	1.99	1.28	0.46 – 10.78	1.94 (1.81, 2.08)	0.055
4 – 7	357 (37.7)	1.94	1.51	0.51 – 45.62	2.00 (1.88, 2.14)	
8 – 11	265 (28.1)	1.99	1.64	0.49 – 27.91	2.03 (1.88, 2.20)	
12 or more	97 (10.0)	1.74	0.94	0.68 – 8.79	1.72 (1.56, 1.90)	
Income class^*b*^						
A and B	348 (36.8)	1.95	1.44	0.48 – 45.62	1.97 (1.85, 2.11)	0.259
C	514 (54.3)	1.97	1.49	0.46 – 13.03	1.98 (1.88, 2.08)	
D and E	85 (8.9)	1.74	1.20	0.51 – 24.49	1.85 (1.61, 2.11)	
Occupation						
Exposed to lead	114 (12.0)	2.48	2.11	0.7 – 45.62	2.65 (2.31, 3.05)	<0.001
Not exposed to lead	834 (88.0)	1.89	1.35	0.46 – 27.91	1.89 (1.82, 1.96)	
Smoking						
Never	498 (52.5)	1.71	1.10	0.46 – 45.62	1.68 (1.59, 1.76)	<0.001
Former	268 (28.3)	2.05	1.57	0.56 – 8.53	2.11 (1.98, 2.25)	
Current	182 (19.2)	2.64	2.18	0.87 – 27.91	2.73 (2.50, 2.98)	
Alcohol Consumption						
Do not drink	584 (61.6)	1.74	1.19	0.46 – 27.91	1.74 (1.67, 1.83)	<0.001
Drink	364 (38.4)	2.26	1.72	0.56 – 45.62	2.38 (2.24, 2.53)	
Systolic blood pressure						
< 140 mm Hg	603 (63.6)	1.88	1.39	0.48 – 27.91	1.86 (1.78, 1.95)	<0.001
≥ 140 mm Hg	345 (36.4)	2.07	1.58	0.46 – 45.62	2.16 (2.02, 2.31)	
Diastolic blood pressure						
< 90 mm Hg	714 (75.3)	1.86	1.35	0.46 – 45.62	1.86 (1.79, 1.94)	<0.001
≥ 90 mm Hg	234 (24.7)	2.19	1.73	0.50 – 24.49	2.31 (2.13, 2.51)	
Hypertension^*c*^						
No	429 (45.3)	1.88	1.46	0.49 – 27.91	1.88 (1.77, 1.98)	0.099
Yes	519 (54.7)	2.00	1.47	0.46 – 45.62	2.04 (1.94, 2.15)	
Glycemia (mg/dL)						
< 100	499 (52.6)	1.85	1.42	0.49 – 27.91	1.91 (1.81, 2.01)	0.278
≥ 100	449 (47.4)	2.00	1.44	0.46 – 45.62	2.03 (1.92, 2.15)	
HDL (mg/dL)						
≥ 50 female ≥40 male	483 (50.9)	2.04	1.68	0.49 – 27.91	2.13 (2.02, 2.26)	0.003
< 50 female <40 male	465 (49.1)	1.84	1.19	0.46 – 45.62	1.80 (1.71, 1.89)	
LDL^*d*^ (mg/dL)						
< 160	767 (83.5)	1.90	1.42	0.46 – 27.91	1.92 (1.84 – 2.00)	0.574
≥ 160	151 (16.4)	2.01	1.36	0.60 – 12.88	2.07 (1.90 – 2.27)	
Total cholesterol (mg/dL)						
< 200	452 (47.7)	1.88	1.48	0.49 – 27.91	1.90 (1.80, 2.01)	0.188
≥ 200	496 (52.3)	1.99	1.36	0.46 – 45.62	2.03 (1.92, 2.14)	
Triglycerides (mg/dL)						
< 150	622 (65.6)	1.89	1.51	0.48 – 27.91	1.94 (1.85, 2.03)	0.296
≥ 150	326 (34.4)	2.01	1.31	0.46 – 45.62	2.02 (1.90, 2.16)	
Body Mass Index^*e*^ (Kg/m2)						
15 - <25	289 (30.8)	1.96	1.74	0.49 – 27.91	2.10 (1.95, 2.26)	0.004
25 - <30	357 (38.1)	2.03	1.40	0.50 – 21.83	2.02 (1.90, 2.14)	
≥ 30	291 (31.1)	1.85	1.15	0.46 – 45.62	1.95 (1.37, 2.78)	

^*a*^ *t*-Student and Kruskal-Wallis test*.* ^*b*^According to ABEP, 2012. Data missing for one participant. ^*c*^According with VI Diretriz Brasileira de Hipertensão. Hypertension defined as systolic blood pressure ≥140 mmHg or diastolic blood pressure ≥90 mmHg or current antihypertensive medication. ^*d*^30 missing data on this variable (n = 918). ^*e*^11 missing data on this variable (n = 937)

Of the 948 participants in the sample, 519 (54.7%) were classified as hypertensive, of whom 398 were classified as hypertensive according to their systolic and/or diastolic blood pressures, and 121 if they self-reported current use of antihypertensive medication. A total of 181 subjects had both systolic and diastolic hypertension, 164 had only systolic hypertension, 53 had diastolic hypertension. Among the 220 untreated subjects, 73 had systolic hypertension only, 39 had diastolic hypertension, and 108 had both systolic and diastolic hypertension. Of those who reported being treated for hypertension (*n* = 299), 91 had only systolic hypertension, 14 had diastolic hypertension, and 73 had both systolic and diastolic hypertension. Participants with hypertension (systolic blood pressure ≥140 mm Hg, diastolic blood pressure ≥90 mm Hg, or self-reported use of antihypertensive medication) had higher blood lead levels (2.04 μg/dL; 95% CI, 1.94–2.15) than those not hypertensive (1.87 μg/dL; 95% CI, 1.77–1.98) (Tables 1 and 2). The results of the Pearson correlation analysis for systolic blood pressure and blood lead levels was *r* = 0.088, p < 0.005, and for diastolic blood pressure the coefficient was *r* = 0.112, p < 0.005.

**Table 2:** Participants characteristics by hypertension status^a^

Characteristics	Hypertension (*n* = 519)	No hypertension (*n* = 429)	All (*n* = 948)	*P* value^*b*^
Age (years)	57.6 (0.46)	50.9 (0.44)	54.5 (0.34)	<0.001
Sex (% male)	42 (0.02)	47 (0.02)	44.5 (0.16)	0.114
Race (% white)	58 (0.02)	61 (0.02)	59 (0.01)	0.375
Education (years of study)	5.62 (0.19)	7.24 (0.21)	6.35 (0.14)	<0.001
Income class (% middle income class)^*c*^	59 (0.02)	48 (0.02)	54 (0.02)	0.001
Smoking				
Former smoker (%)	32 (0.02)	23 (0.02)	28.3 (0.01)	0.001
Current smoker (%)	16 (0.16)	23 (0.02)	19 (0.01)	
Alcohol intake (% drinker)	42 (0.02)	36 (0.02)	38.4 (0.01)	0.055
Total cholesterol (mg/dL)	206.8 (1.83)	200.5 (1.80)	203.9 (1.29)	0.020
Triglycerides (mg/dL)	169.4 (7.65)	129.2 (5.04)	151.2 (4.81)	<0.001
Glycemia (mg/dL)	110.1 (1.52)	98.9 (0.89)	105.0 (0.94)	<0.001
BMI (Kg/m^2^)	28.9 (0.23)	26.9 (0.27)	27.9 (0.18)	<0.001
Blood lead (μg/dL)^*d*^	2.04 (1.94-2.15)	1.87 (1.77-1.98)	1.97 (1.89-2.04)	0.029
Occupation (% lead exposed)	11 (0.01)	13 (0.02)	12.0 (0.01)	0.278

^a^Hypertension defined as systolic blood pressure ≥ 140 mmHg or diastolic blood pressure ≥ 90 mmHg or antihypertensive medication use. ^*b*^Chi-square, Wilcoxon or Kruskal-Wallis test. ^c^According to Associação Brasileira de Empresas de Pesquisa (ABEP, 2012); from 0 (lower income level) to 45 (higher income level). ^d^Geometric mean (95% CI); other results in the table are arithmetic means or percentages (SE)

In unadjusted analysis, participants with elevated systolic and diastolic blood pressure presented higher blood lead concentration (2.16 μg/dL; 95% CI, 2.02–2.31) and (2.31 μg/dL; 95% CI, 2.13–2.51), compared with those with normal systolic and diastolic blood pressure (1.86 μg/dL; 95% CI, 1.78–1.95) and (1.86 μg/dL; 95% CI, 1.79–1.94), respectively. Hypertensive individuals had increased concentrations of total cholesterol, triglycerides and glycemia than those not hypertensive. Controversially, we observed that participants with higher levels of HDL had higher blood lead concentrations than those with decreased HDL (Tables 1 and 2).

In multivariate analysis, there was a weak association of blood lead with both systolic and diastolic blood pressure. After multivariable adjustment, participants in the quartile 4 of blood lead presented 0.06 mm/Hg (95% CI, 0.04–0.09) average difference in diastolic blood pressure comparing with those in quartile 1. For systolic blood pressure, the average difference was almost insignificant (Table 3). Participants in the 90th percentile of blood lead distribution had 0.07 mmHg (95% CI, 0.03 to 0.11) higher diastolic blood pressure compared with those participants in the 10th percentile of blood lead. The geometric mean of blood lead level of participants in the 10th percentile and in the 90th percentile was 0.74 μg/dL (95% CI, 0.71–0.77) and 6.03 μg/dL (95% CI, 5.52–6.58), respectively.

**Table 3:** Change (95% CI) of systolic and diastolic blood pressure by blood lead levels (μg/dL)

	Systolic blood pressure (mmHg)^*^		Diastolic blood pressure (mmHg)	
	Model 1	Model 2	Model 1	Model 2
Blood lead (μg/dL)				
Quartile 1 (≤1.32)	0.00 (reference)	0.00 (reference)	0.00 (reference)	0.00 (reference)
Quartile 2 (1.32–1.93)	−0.00^*^	−0.00^*^	0.04 (0.01–0.05)	0.03 (0.01–0.05)
Quartile 3 (1.93–2.76)	−0.00^*^	−0.00^*^	0.03 (0.01–0.06)	0.02 (0.00–0.05)
Quartile 4 (>2.76)	−0.00^*^	−0.00^*^	0.07 (0.04–0.09)	0.06 (0.04–0.09)
*p*-Trend	<0.001	0.002	<0.001	<0.001

^*^All Confidence Intervals (CI) for systolic blood pressure were −0.00 to -.000

Model 1 was adjusted for sex, age (40–49, 50–59, ≥60), race (white, no white), education (years of study), income (continuous; according to Associação Brasileira de Empresas de Pesquisa, 2012) and antihypertensive medication (yes, no)

Model 2 was further adjusted for continuous total cholesterol (μg/dL), triglycerides (μg/dL), glycemia (μg/dL), smoking status (never, current and former), alcohol intake (yes, no) and body mass index, continuous (kg/m^2^)

The adjusted OR for hypertension comparing the highest to the lowest blood lead quartiles was 2.54 (95% CI, 1.17–5.53). Compared with participants in the 10th percentile of blood lead, participants in the 90th percentile presented higher OR for hypertension (OR: 2.77; 95% CI, 1.41 to 5.46) (Table 4).

**Table 4:** OR (95% CI) of hypertension by blood lead quartiles (μg/dL)

	Model 1	Model 2
Blood lead (μg/dL)		
Quartile 1 (≤1.32)	1.00 (reference)	1.00 (reference)
Quartile 2 (1.32–1.93)	0.22 (0.02–2.97)	0.11 (0.01–1.59)
Quartile 3 (1.93–2.76)	0.58 (0.42–8.22)	0.40 (0.02–6.87)
Quartile 4 (>2.76)	2.28 (1.12–4.66)	2.54 (1.17–5.53)
*p*-Trend	<0.001	0.003
90th – 10th percentile	2.62 (1.40–4.91)	2.77 (1.41–5.46)

Model 1 was adjusted for sex (male, female), age (40–49, 50–59, ≥60), race (white, no white), education (0–3, 4–7, 8–11 and 12 or more years of study), income (classes A/B, C, D/E; according to Associação Brasileira de Empresas de Pesquisa, 2012) and antihypertensive medication (yes, no)

Model 2 was further adjusted for continuous total cholesterol (<200 μg/dL, ≥200 μg/dL), triglycerides (<150 μg/dL, ≥150 μg/dL), glycemia (<100 μg/dL, ≥100 μg/dL), smoking status (never, current and former), alcohol intake (yes, no) and body mass index, continuous (kg/m^2^)

When we further adjusted for occupational status, the results did not significantly change and the overall significance was maintained.

The results of the multivariate analysis when considering only the subjects not taking antihypertensive medication (*n* = 649) were similar to those results of the sample including the whole population. Only a minor centesimal difference was observed for the OR and 95% CI as shown in Tables 3 and 4.

## Discussion

In this study we found a significant association between small increases in blood lead levels and elevations in systolic and diastolic blood pressures. The association of blood lead was +0.06 mm Hg (*p* < 0.001) for diastolic blood pressure, while the association for systolic blood pressure was statistically significant (*p* < 0.05), with no difference observed in the 95% CI, showing a modest increase. However, the OR for hypertension was higher in participants in the quartile 4 of blood lead compared with those in the quartile 1 (OR = 2.54; 95% CI, 1.17–5.53). The geometric mean blood lead concentration of 1.97 μg/dl (95% CI, 1.89–2.04) found in the present study was higher than the concentration of 1.64 μg/dl reported by Bushnik et al. [12], in a survey at national level with the Canadian population aged 40 to 79 years old. These authors reported a significant association between systolic and diastolic blood pressure and lead concentrations for subjects aged 40 to 54 years old and for men, but not with the odds for hypertension [12].

Prospective studies have suggested that there is sufficient evidence to infer a causal relationship of lead exposure with hypertension, besides the modest strength observed in these associations [3, 29–31]. Bone lead is considered a long-term biomarker for lead exposure, representing a cumulative dose in the body, which has been considered a better assessment of lead in epidemiological studies evaluating associations of cumulative exposures with chronic disease. Blood lead represents a short-term biomarker, reflecting recent exposure to the metal, but also provide variability of lead released from the bones [32]. The mobilization of lead from bone is increased during some periods of life, such as aging, pregnancy and after the menopause in women [10, 32, 33]. Studies that measured both bone and blood lead reported that bone lead (patella and tibia bones), was positively associated with hypertension, while blood lead was mostly associated with raised blood pressure [34, 35]. So the associations found between bone lead and current blood pressure indicate that past exposure as well as current exposure to lead are important biomarkers to evaluate blood pressure outcomes.

Studies with general population also found positive associations between blood lead concentrations and elevation on both systolic and diastolic blood pressure [6, 12, 34, 36, 37]. We identified a most significant association of blood lead with diastolic blood pressure (0.06 mmHg; 95% CI, 0.04–0.09), while other studies reported most significant associations of blood lead with systolic blood pressure comparing with diastolic blood pressure [6, 9, 10, 31, 34, 36–38]. This variability is difficult to be enlightened and may not be restricted to intrinsic factors of blood pressure, but also depends on the measurement technique used, the measurer's ability, and other conditions under which the measure will be held [2].

Most recently, Hara et al. [6] reported that blood lead doubling was associated with higher systolic (+0,76 mm Hg; 95% CI, 0.38–1.13) and diastolic blood pressure (+0,43 mm Hg; 95% CI, 0.18–0.68), but not with the odds of prevalent hypertension in analyzing data from NHANES 2003–2010. Besides that, the study reported the results of stratified analysis of blood lead and blood pressure by sex and ethnicity, revealing distinct results regarding each population stratum [6]. Before that, Scinicariello et al. [39] published data from NHANES 1999–2006 and reported that blood lead was significantly associated with higher diastolic blood pressure among white men and women and in black men, while elevated systolic blood pressure was associated with blood lead only in blacks, but not in whites or in Mexican-American participants. Compared with American population, Brazilian adults may be exposed to distinct sources of lead, and the influence of covariates may differ [20]. Besides, although the present study is based on a representative sample of adults, the number of participants did not allow us to perform a fully stratified analysis.

Unlike what has been observed on the hypertensive effects of lead in studies involving workers exposed to high levels of lead, environmental exposure is often asymptomatic and as other toxic effects of lead, the magnitude of raised blood pressure depends on the magnitude of the exposure [2, 3, 5, 11, 40, 41]. Another perspective on the health effects of lead-associated increases in blood pressure is to consider the effect of a unit increase in median BLL on the overall distribution of blood pressure values in a population and the effect on number of persons with blood pressure elevations associated with stroke and other endpoints. This was calculated by Schwartz [42], who reported that a 1 μg/dL increase in mean population blood lead in US white males aged 40–59, distributed over the range of blood pressure values in this population, would increase the numbers of persons at risk for myocardial infarction by 3200 per year, of strokes by 1300 per year, as well as an overall increase in clinical hypertension of 635,000 more cases. Pirkle et al. [43], using the opposite approach, based on a longitudinal analysis of NHANES II data, from 1976 to 1980, in which blood lead levels in white males aged 40 to 59 years decreased by 37%, found that this decrease resulted in 4.7% and 6.7% decrease in fatal and nonfatal myocardial infarctions and strokes, respectively [43].

The inclusion of confounding factors in regression models has been considered very important to control for residuals in such analysis, especially when examining small effects as the influence of lead in blood pressure [10]. The inclusion of inappropriate covariates in regression models can attenuate associations between lead levels and the studied outcomes. Still, the absence of certain variables in the analysis may overestimate the strength of the association [9, 34, 40]. In this study, we included in the analysis risk factors for cardiovascular disease such as smoking, alcohol consumption, sex, age, socioeconomic and demographic variables and BMI to account for covariates that are known to be associated with blood pressure outcomes. Even so, some unmeasured variables not included in the analysis may influence the association of lead and blood pressure as, for example, pre-existing diseases, genetic aspects and renal function [40].

Although not completely elucidated, some mechanisms of lead-related hypertension have been reported, such as: impaired renal function, reduction of nitric oxide (NO) bioavailability, increased oxidative stress, increased activity of the renin angiotensin system and down-regulation of soluble guanylate cyclase. As a consequence of such mechanisms, lead exposure leads to increased vascular tone and induce alterations of the adrenergic system leading to peripheral vascular resistance [3, 44, 45].

Oxidative stress has been described as the main mechanism of lead-induced toxicity. Lead exposure promotes oxidative stress by enhancing the production of reactive oxygen species, and by decreasing antioxidant enzymes activity [45–49]. Even at low levels, lead triggers the occurrence of events that are involved with the development of hypertension and other cardiovascular diseases, which have been consistently reported in studies conducted with animals and humans [50, 51]. The overload of oxygen reactive species leads to tissue injury and dysfunction through direct interaction with cell molecules, and through depletion of the antioxidant system. Lead interaction with biological macromolecules, such as lipids, proteins and DNA, results in disturbances in cell membranes, changing its structure and functioning. Beyond that, lead interferes with the activity of important antioxidant enzymes, such as glutathione, which can interact directly with reactive oxygen species, or can serve as a cofactor for the detoxification reactions in the presence of free radicals [52–55]. The activation of redox-sensitive transcription factors, such as the nuclear factor-κB (NF-κB), which stimulates the production of inflammatory substances associated with renal interstitial inflammation, and changes in the calcium channels function leading to alterations in vascular tone and reactivity, are also important factors contributing for alterations in blood pressure outcomes [51, 56].

Furthermore, lead-induced oxidative stress reduces the availability of NO, which is an endogenous catalyst of several biochemical processes with an important role in cardiovascular system regulation, leading to endothelial dysfunction [41]. Barbosa Jr et al. [57] reported that lead exposure exerted a significant inhibitory effect on NO production, as shown by analyses of nitrite concentrations in adults living in the Southeast region of Brazil, suggesting that this biological mechanism is possibly related to the increased cardiovascular risk associated with lead exposure.

Some positive points to be highlighted in our study are the census based and the spatial analysis used to enroll participants. Besides that, the study included the measurement of multiple anthropometric and socioeconomic characteristics and the standardization of blood pressure measurements (≥3 readings) based on Brazilian Guidelines, taken by trained researchers. However, some limitations of this study are the cross-sectional design that does not allow causal inferences about the association of blood lead and blood pressure; and the fact that we may not have measured all the potential confounders of the relationship between hypertension and lead exposure.

Finally, we found that participants in the highest quartile of blood lead had an OR for hypertension of 2.54 (95% CI, 1.17–5.53), compared with those in the lowest quartile, as well as participants in the 90th percentile of blood lead presented higher OR for hypertension than those in 10th percentile. Differences observed among the results of other studies may be related to methodological issues, as bone lead measurement as a marker of lead exposure compared with blood lead and to stratified statistical analysis, which have shown that distinct risk factors for lead exposure exist among these populations. Although education and income have been considered significant predictors of blood lead levels in Americans, the historically difference observed among income level has been decreasing. Furthermore, some Pb-related outcomes, including blood pressure outcomes, may vary by race/ethnicity, providing some evidence that some groups may be at higher risk of developing some diseases [7, 44, 58]. In Brazil these characteristics may have specific associations with both blood lead levels and hypertension, indicating that more studies with Brazilian adults are necessary to evaluate Pb-related health outcomes, as well as to provide an evaluation of blood lead levels in the general population.

## Conclusions

We found a positive association between blood lead and diastolic blood pressure, and a significant association of blood lead and hypertension in Brazilians aged 40 years or older, living in southern Brazil. Our results add knowledge about the health risks associated with current environmental exposures to lead in Brazil, and encourage efforts to prevent lead exposure in the general population.

## Abbreviations

ABEP: Associação Brasileira de Empresas de Pesquisa
AP-1: Activator protein-1
BLL: Blood lead levels
BMI: Body mass index
CCEB: Economic Classification Criterion Brazil
CI: Confidence interval
CNAE: National Classification of Economic Activities
DALYs: Disability adjusted life years
DBP: Diastolic blood pressure
HDL: High-density lipoprotein
ICP-MS: Inductively coupled plasma mass spectrometry
LDL: Low-density lipoprotein
NF-κB: Nuclear factor-κB
NHANES: National Health and Nutrition Examination Survey
NIST: National Institute of Standards and Technology
NO: Nitric oxide
OR: Odds ratio
Pb: Lead
SBP: Systolic blood pressure
